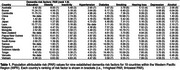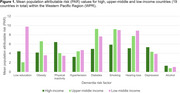# Country specific recommendations for reducing dementia risk in the Western Pacific Region

**DOI:** 10.1002/alz70860_101857

**Published:** 2025-12-23

**Authors:** Claire V Burley, Hamid R Sohrabi, Jennifer Dunne, Blossom CM Stephan

**Affiliations:** ^1^ Dementia Centre of Excellence, Curtin University, Perth, Western Australia, Australia; ^2^ School of Psychology, Murdoch University, Murdoch, Western Australia, Australia; ^3^ Murdoch University, Murdoch, Western Australia, Australia; ^4^ Curtin University, Perth, Western Australia, Australia

## Abstract

**Background:**

The Western Pacific Region (WPR) currently has the third‐highest prevalence of dementia globally and is projected to have the highest prevalence by 2050, with >76 million cases. The 2024 Lancet Commission on Dementia suggests that tackling 14 modifiable risk factors could prevent 45% of cases globally^1^. However, the WPR is socioeconomically and culturally diverse, requiring region‐specific strategies to address dementia effectively.

**Method:**

The population attributable risk (PAR) for nine (out of 14) Lancet Commission factors was calculated for 19 WPR countries using relative risk values from the Lancet Commission and published risk factor prevalence data. The nine factors comprised low education, hearing loss, depression, physical inactivity, diabetes, smoking, hypertension, obesity, and alcohol misuse. Mean PAR values were also calculated for high‐income, upper‐middle income, and low‐middle income WPR countries.

**Results:**

PAR values varied across WPR countries (Table 1). The greatest differences were observed for education (18.5%) and obesity (12.0%) and smallest for depression (3.3%) and alcohol (2.1%). In low‐middle income countries, the highest mean PAR value was for education (9.75%) (Figure 1). By contrast, the highest values in middle‐high and high‐income countries were for diabetes (9.21%) and inactivity (6.42%). Smoking had the second highest mean PAR value in all three groups (range: 5.9‐9.2). Hearing loss and obesity also ranked highly across all groups.

**Conclusion:**

Country‐specific population‐level strategies are urgently needed to effectively reduce dementia risk. Low‐middle income countries will benefit most from interventions and policies that improve access to education; middle‐high income countries from targeting diabetes, and high‐income countries from targeting physical inactivity. Targeting smoking and hearing loss will benefit the entire WPR region. Strategic partnerships between academics, policy makers and industry will ensure appropriate resources are directed to where they will have the most impact.

**References**

Livingston G et al. (2024). Dementia prevention, intervention, and care: 2024 report of the *Lancet* standing Commission. The Lancet, Volume 404, Issue 10452, 572‐628.

Clarke AJ et al. (2024). Risk factors for the neurodegenerative dementias in the Western Pacific region. The Lancet Regional Health – Western Pacific, Volume 50,101051